# Polymer tube nanoreactors *via* DNA-origami templated synthesis†
†Electronic supplementary information (ESI) available: Details of DNA origami preparation and purification. Details of stepwise creation methods of polymer coated DNA tubes. Details of AFM, TEM, DLS, and agarose gel electrophoresis. See DOI: 10.1039/c7cc09620h


**DOI:** 10.1039/c7cc09620h

**Published:** 2018-02-21

**Authors:** Yu Tokura, Sean Harvey, Xuemei Xu, Chaojian Chen, Svenja Morsbach, Katrin Wunderlich, George Fytas, Yuzhou Wu, David Y. W. Ng, Tanja Weil

**Affiliations:** a Max Planck Institute for Polymer Research , Ackermannweg 10 , 55128 Mainz , Germany . Email: Weil@mpip-mainz.mpg.de ; Email: David.Ng@mpip-mainz.mpg.de; b Inorganic Chemistry I , Ulm University , Albert-Einstein-Allee 11 , 89081 Ulm , Germany . Email: tanja.weil@uni-ulm.de; c Hubei Key Laboratory of Bioinorganic Chemistry and Materia Medica , School of Chemistry and Chemical Engineering , Huazhong University of Science and Technology , Luoyu Road 1037 , 430074 Hongshan , Wuhan , P. R. China

## Abstract

We describe the stepwise synthesis of precise polymeric objects programmed by a 3D DNA tube transformed from a common 2D DNA tile as a precise biotemplate for atom transfer radical polymerization. The catalytic interior space of the DNA tube was utilized for synthesizing a bio-inspired polymer, polydopamine.

## Abstract 2

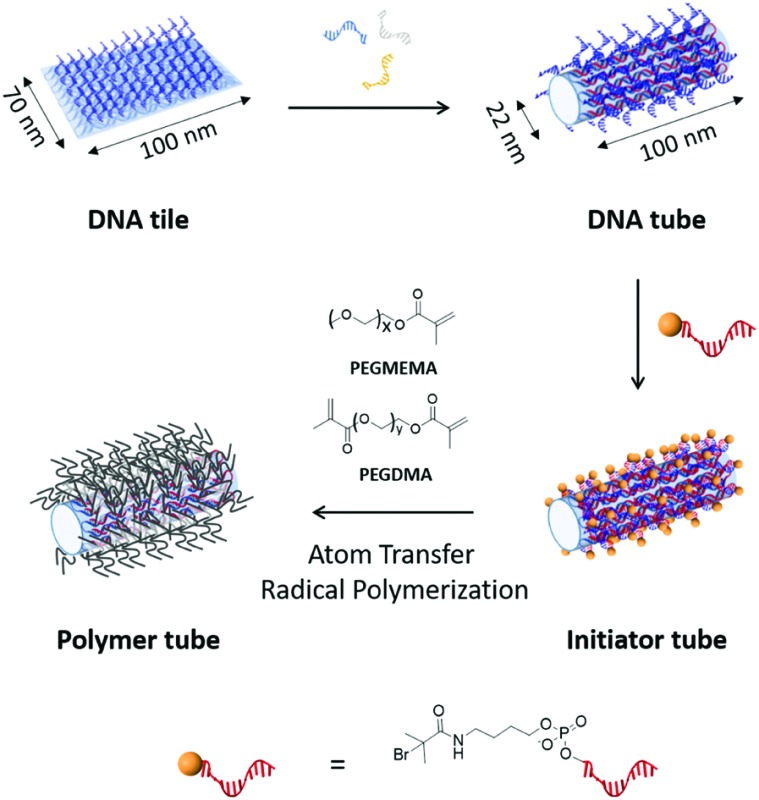
We describe the stepwise synthesis of precise polymeric objects programmed by a 3D DNA tube transformed from a common 2D DNA tile as a precise biotemplate for atom transfer radical polymerization.

## 

Three-dimensional engineering of nanomaterials with precise control over sizes, shapes and functionalities represents the epitome of nanotechnology with far-reaching applications from materials science to personalized medicine. From a molecular perspective, the formation of DNA origami and associated strategies^1^ to program functionalities with absolute positioning remains an unrivalled technology even in the years to come. Nonetheless, as a standalone material, polyanionic DNA is lacklustre as it generally requires a cocktail of Ca^2+^/Mg^2+^ fortified buffers to remain hydrolytically stable.^2^ Transferring the structural information of DNA origami to polymeric materials would provide access to unprecedented 3D architectures of customized material properties and presumably improved stability compared to the DNA origami scaffold.^3^

Methodically, the emergence of DNA nanotechnology and, in particular, the “DNA origami” technique is supported by computer-aided design. As a result, precise DNA nano-objects between 20 and 100 nm can be created with near-limitless flexibility.^4^ This significant advantage is compounded by the capability to designate any specified positions on the 3D architecture for further modifications, which thus represents the core of nanoscale programmability. Using these principles, a wide range of biotechnological applications including multi-enzyme cascade systems,^5^ drug delivery carriers,^6^ and artificial ion channels^7^ have been developed. Besides these biotechnological advances, the impact of DNA origami in shaping organic^8^ and inorganic^9^ nanoarchitectures has also been increasingly investigated. In this communication, we designate a DNA tube as a shape-persistent 3D framework to direct and pattern the *in situ* growth of different polymers in an orthogonal fashion. By decorating the outer surface of the DNA tube with initiators for atom transfer radical polymerization (ATRP), a templated architecture was achieved *via* polymerization with cross-linking (Fig. 1). As a proof of concept to demonstrate 3D engineering, we functionalize the interior space with multiple DNA-based catalytic moieties (DNAzyme) as reaction sites for the oxidative polymerization of dopamine. By accomplishing these strategies in sequence, we show the versatility of DNA origami as a platform to exert orthogonal control over both the shape and cross-sectional components of a nanostructure.

**Fig. 1:**
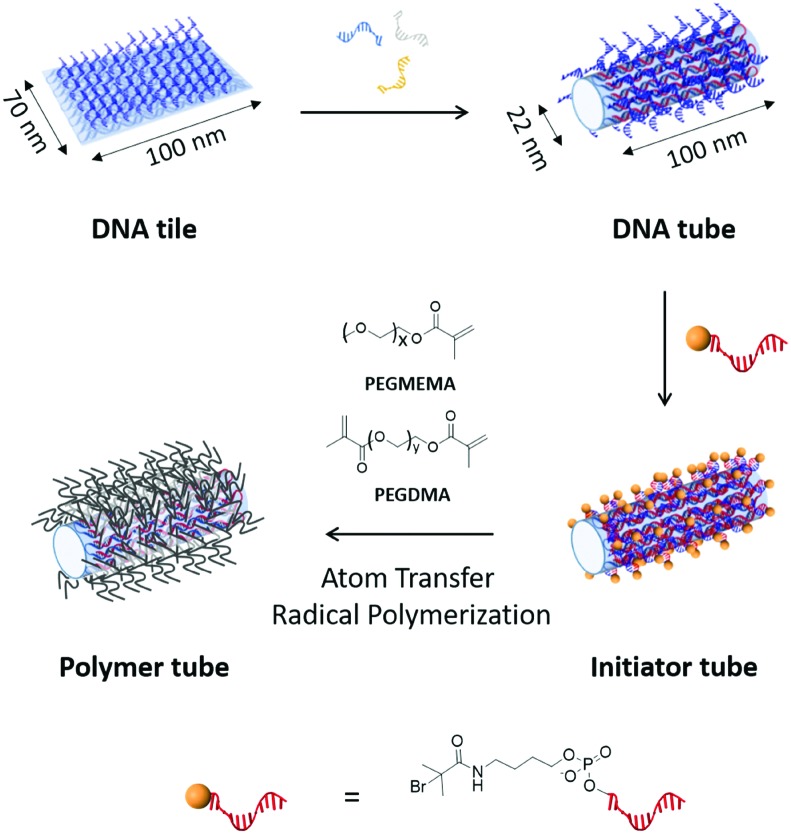
DNA tiles decorated with multiple single-stranded DNA handles are transformed to DNA tubes by applying folding DNA strands. After equipping the outer surface of DNA tubes with ATRP-initiator moieties, polymers are grown on the surfaces to form a polymer tube. Bottom: The molecular structure of initiator modified DNA is represented. The 5′ end of DNA (red) is modified with bromoisobutyrate (yellow sphere).

First, the DNA tube was constructed in a stepwise fashion, starting from a DNA tile (70 nm × 100 nm with 2 nm thickness),1*a* by annealing a scaffold DNA strand (M13mp18) and 210 staple DNA strands. To functionalize its surface with ATRP initiator molecules in the subsequent step, 169 staple DNA strands were modified with an additional single-stranded DNA (ssDNA) sequence as a DNA handle (Fig. S1 and Table S2, ESI†).^10^ All DNA handles are designed to appear and fully cover only one surface of the DNA tile. Thereafter, the transformation of the DNA tile to the DNA origami tube (22 nm in diameter and 100 nm in length) was accomplished by applying additional 16 DNA sequences (folding DNA strands), which connect the two long edges of the DNA tile to form the DNA tube (Fig. 1, upper part).^10^ Excessive amounts of folding DNA strands were removed by PEG-induced precipitation.^11^

Atomic force microscopy (AFM) and transmission electron microscopy (TEM) revealed the successful conversion from the DNA tile into DNA tubes (Fig. 2 and Table 1). From AFM, the dimensions of the DNA tube in 1× (Tris)-acetate–ethylenediamine tetraacetic acid (EDTA) buffer with 12 mM MgCl_2_ (1× TAE/Mg buffer) were determined to be 36 ± 6 nm in diameter and 5 ± 0.7 nm in height. The discrepancy (+14 nm in diameter and –17 nm in height) between these measured dimensions in AFM and the theoretical calculations could be attributed to a structural distortion of the DNA tube by the strong attractive interaction between the DNA tube surface and the mica surface *via* Mg^2+^ bridging. In addition, agarose gel electrophoresis (AGE) and dynamic light scattering (DLS) studies were conducted. In AGE, the band shift was observed after the transformation (Fig. 2D) and DLS revealed an increase of the hydrodynamic radius (*R*_h_) from 55 ± 3 nm (DNA tile) to 73 ± 6 nm (DNA tube) (Fig. S2, ESI†). The DNA tile and the DNA tube were investigated using static light scattering (SLS) to determine the radius of gyration: *R*_g_ = 54 ± 2 nm for the DNA tile and *R*_g_ = 83 ± 2 nm for the DNA tube (lower inset of Fig. S2, ESI†). From *R*_g_ and *R*_h_, the shape factor, *R*_g_/*R*_h_, varies for different particle architectures and geometries and is a valuable parameter for the determination of the shape.^12^*R*_g_/*R*_h_ = 0.98 for the DNA tile and 1.14 for the DNA tube were obtained, noting that the shape changed. Next, the obtained DNA tube was equipped with 169 initiator molecules for homogeneously covering the outer surface of the DNA tube with the polymer shell. Bromoisobutyrate was modified to 5′ of the ssDNA strand^13^ and subsequently hybridized onto the multiple ssDNA handles on the surface of the DNA tube. The surface initiated ATRP on the DNA tube was conducted using poly(ethylene glycol) methyl ether methacrylate (PEGMEMA, average *M*_n_: 300) as the monomer due to its amphiphilic character, which is widely known to stabilize sensitive biomolecules.^14^ In order to achieve a tight polymer network, PEG dimethacrylate (PEGDMA, average *M*_n_: 750) was added as a crosslinker. The surface initiated ATRP was conducted as reported previously.3*b* Briefly, 20 μL reaction volume consisting of a 1 : 665 ratio of the DNA origami macroinitiator (50 nM) and the sacrificial initiator (33 μM), PEGMEMA, PEGDMA, CuBr_2_ and tris(2-pyridylmethyl)amine (TPMA) were combined. The reaction mixture was degassed *via* the freeze–pump–thaw method, followed by continuous slow addition of ascorbic acid to generate the reactive catalyst. After 2 h, the product was purified by the PEG-induced precipitation and characterized by AFM, TEM, AGE (Fig. 2) and DLS (Fig. S3, ESI†).

**Fig. 2:**
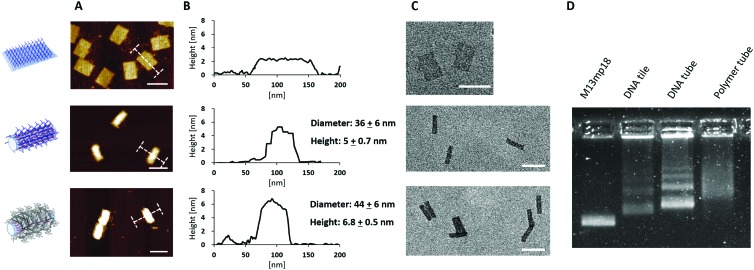
(A) AFM images of DNA tile (upper), DNA tube (middle) and polymer tube (bottom) respectively measured in liquid (1× TAE/Mg buffer). (B) Height profile of each construct depicted with I-bar in (A) and dimensions of DNA tubes and polymer tubes. (C) TEM images of each construct. Since uranyl formate staining for DNA origami does not visualize the coated polymer shells, there were no obvious structural changes observed between DNA tubes and polymer tubes. However, polymer tubes showed side-to-side stacking behaviour, which was not seen for the DNA tubes. (D) Agarose gel electrophoresis of each DNA construct stained with SYBR Gold. The band was shifted after transformation from the tile to the tube and after polymer formation. All the images were measured with purified samples. Scale bars in (A) and (C) are 100 nm.

**Table 1:** Summary of dimensions of the DNA tile, DNA tube and polymer tube from theoretical, AFM, and DLS

Construct		Theoretical (nm)	AFM (nm)	DLS
DNA tile	L	100	99.0 ± 2.2	55 ± 3
	W	70	78.0 ± 4.0	
	H	2	3.1 ± 0.1	

DNA tube	L	100	97.0 ± 4.9	73 ± 6
	W	22	36.0 ± 6.0	
	H	22	5.0 ± 0.7	

Polymer tube	L	—	91.0 ± 6.4	122 ± 13
	W		44.0 ± 6.0	
	H		7.0 ± 0.5	

AFM measurements of the polymer coated DNA tubes (polymer tubes) revealed an increase of both the diameter (+8 nm) and the height (+2.0 nm) compared to the DNA tube due to the surrounding polymer shell. TEM images revealed no obvious structural differences, which is conceivable since the polymer shell could not be stained by uranyl formate.^15^ Nonetheless, TEM images of the polymer tubes revealed the interesting phenomenon that some tube structures showed a lateral stacking (Fig. S4, ESI†). This was also reported for the electrostatic binding of positively charged polylysine to a negatively charged DNA nanostructure15*a* serving as an indication for successful polymer coating. AGE of the polymer tube revealed a further band shift to the slower mobility region possibly due to a reduction in negative surface charges and increased molecular weight by the polymer shell (Fig. 2D). Based on DLS and SLS experiments on dilute solutions of polymer tubes, the increased *R*_h_ and *R*_g_, 122 ± 13 nm and 108 ± 3 nm (Fig. S3, ESI† and Table 1), are most likely due to the grown polymer layer and the altered hydration shell around the polymer tube. The shape factor *R*_g_/*R*_h_ = 0.88 is even smaller than those for both the DNA tile and the tube, which clearly implies a form change upon polymer tethering and a compact polymer structure. Furthermore, other higher-ordered interactions such as the increased hydration sphere or large aggregation of the entire construct were not observed. The stability of the polymer tubes against nuclease digestion was evaluated using a dsDNA-intercalating dye, SYBR Safe, as a reporter molecule (Fig. S5, ESI†). In the presence of 50 mU nucleases, the emission of SYBR Safe in the DNA tiles and tubes decreased to 20% and 26% of the original signals, respectively. Under the same conditions, the polymer tube still retained about 60– 70% fluorescence intensity indicating that the polymer shell protected the DNA tube from nuclease digestion. It should be noted that the ends of the DNA tube are open and in principle still accessible for nuclease digestion, which could explain the observed decrease in fluorescence intensity by about 30%.

A major advantage of the grafting-from strategy is the spatial control over the coating area and the high density of the polymer chains. For 3D nanoscale engineering, the polymer tube provides a unique opportunity to introduce further functionalities within the interior cavity. DNAzymes were introduced into the DNA tube to serve as reaction centers. Guanine-rich sequences adopt a unique secondary structure called G-quadruplex (G4) composed of stacked square planar guanine tetrads.^16^ The thus formed G4 structure can accommodate hemin to activate its catalytic activity mimicking horseradish peroxidase (HRP),^17^*i.e.* acting as a redox catalyst. We functionalized the inner surface of the DNA tube with twenty G4 moieties and a polymer shell was introduced as described above (Fig. 3b and Fig. S6, ESI†). The initial G4-DNA tube revealed similar dimensions (diameter: 37 ± 4 nm, height: 7.2 ± 1.0 nm, Fig. 3c) to the DNA tube itself. Polymerization *via* ATRP was subsequently conducted and the G4 incorporated polymer tube (G4-polymer tube) was obtained with a larger diameter of 55 ± 10 nm and a height of 11 ± 1.9 nm (Fig. 3d and Table S1, ESI†).

**Fig. 3:**
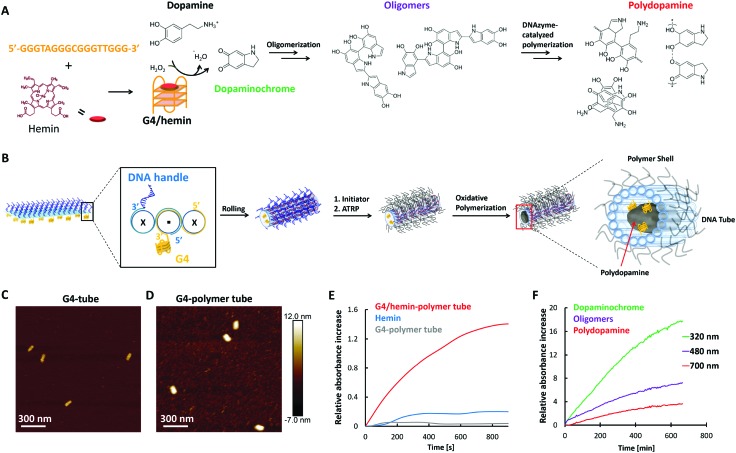
(A) Scheme of G4/hemin-based DNAzyme formation and the proposed mechanism of polydopamine formation. (B) Schematic illustration for the programmed and orthogonal initiation of ATRP and polydopamine formation. The G4 moieties were incorporated with the corresponding staple DNA strands being split into two strands: G4-modified strands (yellow) and DNA handle strands (blue). Both modifications were introduced at 3′ of the DNA strand (Fig. S6, ESI†). (C and D) AFM images of G4 incorporated DNA tube before and after polymer coating. (E) ABTS assay of G4/hemin-polymer tube. (F) Kinetics of the polymerization of dopamine initiated by the G4/hemin-polymer tubes.

The activity of the G4/hemin DNAzyme loaded into the polymer tube was assessed following the oxidation of 2,2′-azino-bis(3-ethylbenzothiazoline)-6-sulphonic acid (ABTS) in the presence of hydrogen peroxide. Its positive catalytic activity was compared against free hemin as well as the unloaded G4-DNA polymer tube without hemin as controls (Fig. 3e), and the catalytic activity of the G4/hemin DNAzymes inside the polymer tubes was clearly demonstrated. Moreover, upon nuclease addition (50 mU, >3500 s observation time), 80% of the DNAzyme activity was retained suggesting that the respective catalytic domains were protected from nuclease degradation (Fig. S7, ESI†). Subsequently, the peroxidase activity of the G4/hemin DNAzyme was exploited to initiate polymerization of dopamine within the polymer tube. Polydopamine is a highly crosslinked natural polymer of high structural rigidity that can be obtained *via* G4/hemin DNAzyme-catalyzed oxidative polymerization on DNA origami as reported by us.3*a* G4/hemin DNAzyme oxidizes dopamine to dopaminochrome, one of the key intermediates for polydopamine formation.^18^ The reaction kinetics and the formation of polymerization intermediates (dopaminochrome and oligomers) and polydopamine formation were monitored using absorbance spectroscopy (Fig. 3f and Fig. S6, ESI†). In addition, we have shown previously that the G4/hemin DNAzyme functions as a specific anchor and that the polymerization of dopamine in a free solution does not occur.3*b* As such, the opposing placements of the G4 catalytic sites with respect to the ATRP initiators have successfully provided the basis of spatial control between the inner and outer space of the DNA tube.

In conclusion, we have demonstrated the construction of precisely templated DNA-polymer tubes with interfacial orthogonality towards nanoscale engineering. The well-known 2D DNA tile structure from Rothemund was transformed into a 3D DNA tube decorated with multiple ssDNA handles outside, while the interior space was functionalized with G4/hemin-based DNAzymes. The DNA handles immobilized initiator molecules where surface initiated polymerization enables the growth of a densely crosslinked polymeric shell. The internal G4 was loaded with hemin and transformed into DNAzymes that initiate dopamine polymerization. By integrating two mechanistically different polymerizations while providing precision control, the proposed strategy serves as an elegant approach towards 3D polymer engineering on the nanoscale.

We acknowledge support by the European Research Council (ERC, Synergy Grant 319130-BioQ and ERC, Advanced Grant 694977-SmartPhon) and the BMBF (Biotechnologie 2020+ initiative, “Selekomm” project). C. C. acknowledges support from the Promotionskolleg Pharmaceutical Biotechnology funded by the state of Baden-Württemberg through the Ministerium für Wissenschaft, Forschung und Kunst Baden-Württemberg. We thank Dr Rüdiger Berger for critical reading of the manuscript and valuable comments. Open Access funding provided by the Max Planck Society.

## Conflicts of interest

There are no conflicts to declare.

## Supplementary Material

Supplementary information
