# Supplementary material for: Polymer tube nanoreactors via DNA-origami templated synthesis
Source: Chem Commun (Camb). 2018 Feb 21;54(22):2808–11. doi: 10.1039/c7cc09620h (PMC5885267; doi:10.1039/c7cc09620h)
Supplement: Supplementary file 1 [file CC-054-C7CC09620H-s001.pdf]

Electronic Supplementary Material (ESI) for ChemComm.
This journal is © The Royal Society of Chemistry 2018

Supporting information

Polymer tube nanoreactors by DNA-origami templated synthesis

Yu Tokura,[ab] Sean Harvey,[b] Xuemei Xu,[c] Chaojian Chen,[ab] Svenja Morsbach,[b] Katrin Wunderlich,[b] George Fytas,[b] Yuzhou Wu,[bc*] David Y.W. Ng,[b*] and Tanja Weil[ab*]

Table of Contents

General methods 3

Materials and Instruments

Fabrication of DNA tile with multiple DNA handles

Transformation to DNA tube

Synthesis of DNA tube / initiator

Surface initiated atom transfer radical polymerization

Atomic force Microscopy (AFM)

Agarose gel electrophoresis

Transmission electron microscopy (TEM)

Dynamic and static light scattering (DLS and SLS)

Nuclease digestion assay

Kinetics of polydopamine formation on G4/hemin DNA nanotile

Supplementary figures and tables 7

Figure S1 DNA tile

Figure S2 Relaxation functions Cvv(q,t) for the translational diffusion dynamics in aqueous solution of DNA tile (black filled squares) and DNA tube (red filled circles) at 20°C at a scattering wave vector (q=0.009 nm-1) represented by a stretched exponential function (solid lines)

Figure S3 Normalized field correlation functions Cvv(q,t) at a scattering wave vector q=0.009 nm-1 (black filled squares) and q=0.024 nm-1 (red filled circles) for the translational diffusion dynamics in aqueous solution of polymer coated DNA tube at 20°C

Figure S4 TEM image of stacking polymer tube

Figure S5 Stability of different DNA origami structure against nuclease.

Figure S6 DNAzyme-incorporated DNA tile

Table S1 Summary of dimensions of the DNA tile, DNA tube and polymer tube from theoretical, AFM, and DLS

Figure S7 ABTS activity of DNAzymes in polymer tubes before / after nuclease addition (50 mU)

Table S2 Detail of staple DNA sequences

References 22

General methods

Materials and instruments

All solvents and reagents were purchased from commercial sources and were used without further purification.

All solvents and chemicals were purchased from commercial sources and were used without further purification. DNA staple strands and ATRP initiator modified DNA (DNA-initiator) were either synthesized by 12-Column DNA Synthesizer from POLYGEN GmbH and purified by Agilent 1260 Infinity HPLC system with Agilent Eclipse XDB-C18 column or purchased from Sigma-Aldrich. Agarose gel electrophoresis was performed using Bio-Rad Mini-Sub Cell GT horizontal electrophoresis system. Bio-Rad MyCycler™ Thermal Cycler was used for annealing of MP13mp18

phage DNA and DNA staple strands to form DNA origami. Concentration of DNA origami was determined by Spark ® 20M with Nanoquant plate™.

Fabrication of DNA tile with multiple DNA handles

DNA tile with multiple DNA handles was assembled respectively by mixing M13mp18 phage DNA of 7k nt with desired staple strands and modified staple strands in 1 × TAE / Mg buffer (5 mM Tris, 1 mM EDTA, 5 mM NaCl, and 12 mM $MgCl_2$, pH 8.0) and annealing from 65 °C to 20 °C over 2 h, followed by purification with polyethylene glycol (PEG) precipitation method.[1] Briefly, the DNA tile was treated with 15% PEG(8000) (w/v), 5 mM Tris, 1 mM EDTA, and 505 mM NaCl. The solution was mixed well and centrifuged at 12000 g, at room temperature (RT) for 25 min. The supernatant was removed and the pellet was dissolved in 1 × TAE / Mg buffer. The same procedure was conducted twice to remove all the remaining staple DNA sequences.

Transformation to DNA tube

To DNA tile (0.5 pmol) solution was added a set of folding DNA strands (250 pmol each) and the mixture was incubated at 32 degree for overnight. The obtained DNA tube was pourified again with PEG precipitation method.

Synthesis of DNA tube / initiator

DNA tubes (0.75 pmol in 1 × TAE / Mg buffer) were incubated with DNA-initiator [2] (1 nmol in 0.2 μL aqueous solution) at room temperature for 4 h and they were used as DNA tube / initiator without any purification. 1.5 μL of 20 × TAE / Mg buffer was added to the reaction mixture to keep the constant concentration of Mg^{2+}. The excess amount of DNA initiators serves as sacrificial initiator in the ATRP reaction.

Surface initiated atom transfer radical polymerization

A catalyst stock solution of $CuBr_2$ (0.45 mg, 0.002 mmol) and Tris (2-pyridylmethyl) amine (TPMA, 4.64 mg, 0.016 mmol) were prepared in 100 μL of N,N-Dimethylformamide (DMF) and MilliQ water (1 to 1 volume) mixture. The ascorbic acid stock solution, which can generate the active catalyst species, was prepared at 5 mM in 50 mM NaCl, followed by degassing with argon bubbling for 40 mins. To conduct the polymerization reaction, PEGMEMA (Mn = 300l), PEGDMA (Mn = 750), DNA tube / initiator, the catalyst stock solution (1 μL), 20 x TAE buffer (4 μL) were added with the ratio of PEGMEMA: PEGDMA: Initiator = 7200: 800: 1. The reaction solution was degassed with three freeze–pump–thaw cycles and then filled with argon. Ascorbic acid solution (36μL) was feed into the reactor by a syringe pump at the speed of 0.3 μL/min under stirring. The pump was turned off after 2 h and the reactor was incubated for another 4 h. The reaction mixture

after polymerization was purified by 15 % PEG precipitation to obtain the polymer tube.

Atomic force Microscopy (AFM)

Imaging was performed with a Bruker Dimension FastScan Bio AFM equipped with the ScanAsyst mode. The sample solution was deposited onto freshly cleaved mica surface, and left for 5 min at room temperature to allow adsorption of the DNA origami structures. After addition of 70 µL of 1 x TAE / Mg buffer, the sample was scanned with the scan rates between 1 and 3 Hz. Several AFM images were acquired at different areas of the mica surface to ensure the reproducibility of the results. All images were analyzed by using the NanoScope Analysis 1.50 and Gwyddion 2.38 software.

Agarose gel electrophoresis

5 uL of sample (1.5 nM) was mixed with 1 uL of 6 x loading buffer and run with 0.8 % agarose gel in 0.5 x TBE / Mg for 120 minutes in ice bath. After running, the gel was stained by SYBR Gold for 30 minutes and the image was taken by G: Box Chemi (Syngene).

Transmission electron microscopy (TEM)

5 uL of sample (1 nM) was applied on carbon coated copper grid with hydrophilic treatment. After 10 minutes incubation, the remaining solution was removed and the sample grid was stained with 2 % uranyl formate solution for 20 seconds. The stained grid was washed with filtered water for three times and dried in air. Imaging was done with JEOL 1400 instrument and obtained images were analyzed by ImageJ software.

Dynamic and static light scattering (DLS and SLS)

Light scattering measurements were performed with an ALV/CGS3 compact goniometer system with a He/Ne laser (632.8 nm), ALV/LSE-5004 multiple-tau full-digital correlator and ALV5000 software. For temperature controlled measurements, the light scattering instrument was equipped with a thermostat from Julabo. Measurements were performed at 20 °C at 13 angles ranging from 30° to 150°. All DNA origami solution samples were adjusted to a concentration of 3.5 nM in in TAE / Mg / K (0.3 mM Tris, 0.2 mM acetic acid, 0.06 mM EDTA, 0.6 mM MgCl2, 10 mM KCl, pH 5.3). The solutions were then filtered through Hydrophilic Durapore® filters with a pore size of 0.22 µm (Merck Millipore, Billerica, USA) and transferred into dust-free quartz light scattering cuvettes (Hellma, Müllheim, Germany), which were cleaned before in sagewith acetone in a Thurmont-apparatus. The scattering wave vector q is defined as $q = \frac{4\pi n}{\lambda} sin\frac{\theta}{2}$ with n=1.333 being the water refractive index. The relaxation function, C(q,t) = $[G(q,t0-1]^{1/2}$ computed from the

experimental scattering intensity autocorrelation function G(q,t) was represented either by an inverse Laplace transform (ILT) analysis using the CONTIN algorithm.

In dilute solutions, the relaxation rate $\Gamma(q)=1/\tau(q)$ is usually diffusive defining the diffusion coefficient $D=\Gamma(q)/q^2$. For species with small size R i.e., , both the scattering intensity I(q) and $D=D_0$ are q-independent with $I \sim cM$ and $D_0=k_BT/(6\pi\eta_0R_h)$ where c, M, R_h, η_0, k_B, and T are the probed species concentration, its molecular weight and hydrodynamic ratio, the solvent viscosity, the Boltzmann constant and the absolute temperature, respectively. For $qR \sim 1$, both I(q) and D(q) depend on q defining the probing length ($2\pi/q$). The former, known as the form factor, yields (at low qR_g) the radius of gyration R_g,

$$I(q)^{-1}=I(0)^{-1}(1+q^2R_g^2/3) \qquad (1)$$

whereas the effective D is given by,

$$D = D_0(1 + Aq^2) \qquad (2)$$

with A is a parameter characterizing the shape of the diffusing species.

Nuclease digestion assay

DNA tile, DNA tube, and polymer tube were labeled with 0.5 x SYBR-safe solution by during 30 min of incubation. Different amounts (0-50 mU) of nuclease were added to the labeled DNA origami structures and incubated at 37 degree for 30 min. The fluorescence intensity of SYBR-safe was checked by Spark ® 20M with Nanoquant plate™ and compared to the sample, to which no nuclease was added.

ABTS assay

To 0.3 nM G4-DNA tile, G4-DNA tube, and G4-polymer tube in the buffer composition (97 uL, 20 mM Tris, 1 mM EDTA, 12 mM $MgCl_2$, pH 5.3 by addition of acetic acid) was added 1 uL of 100 nM hemin. The assay was performed by mixing the hemine added DNA origami solution with1 uL of freshly prepared 50 mg/ml ABTS solution and 1 uL of 0.1M H_2O_2. Immediately after H_2O_2 addition, the absorbance spectrum was measured by using a Tecan Spark® 20M plate reader.

Kinetics of polydopamine formation on G4/hemin DNA nanotile

G4-DNA tube (3.5 nM) in TAE / Mg / K (0.3 mM Tris, 0.2 mM acetic acid, 0.06 mM EDTA, 0.6 mM MgCl2, 10 mM KCl, pH 5.3) was mixed with hemin (70 nM) for 30 min at rt. 98 μL of the solution was added to a 384 well UV transparent plate. To G4/hemin DNA nanotile solution was added 1 uL of a freshly prepared 1M dopamine solution and 1 uL of 1M H2O2. Immediately after H_2O_2 addition, the absorbance spectrum was measured every 5 minutes for a duration of 12 hours using a Tecan Spark® 20M plate reader.

Supplementary figures and tables

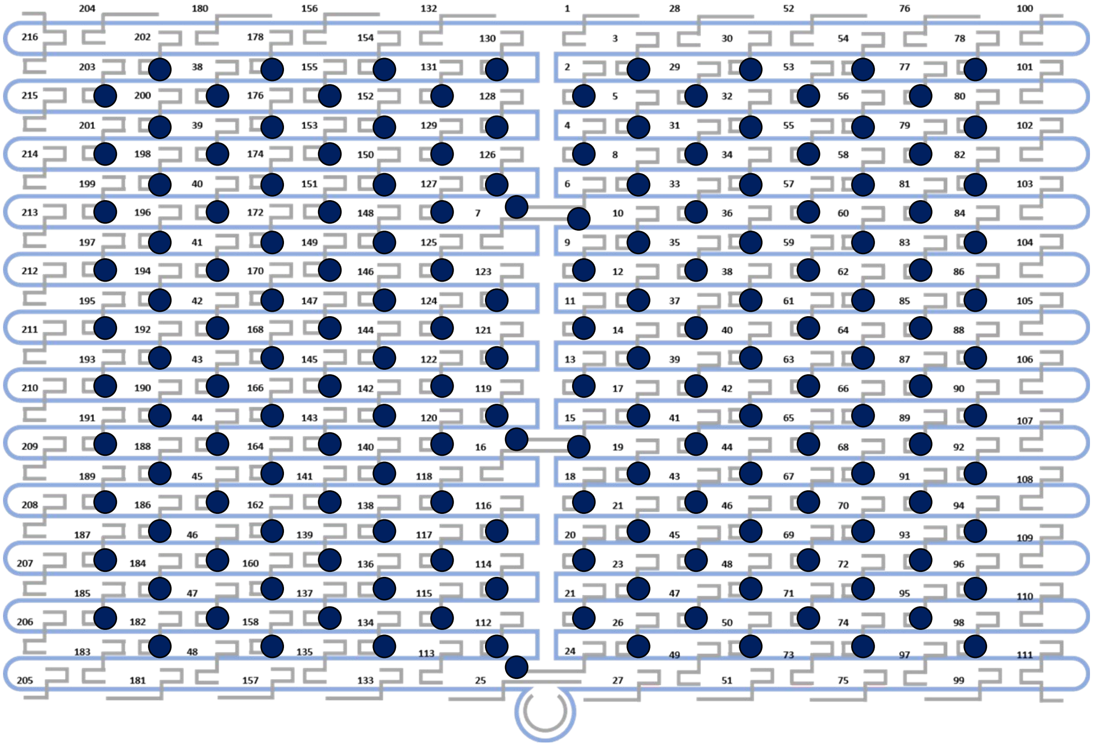

Figure S1 DNA tile. The design of DNA tile[3] and the position of DNA handles to attach ATRP initiator moieties (dark blue circle) chosen from Cadnano software.[4] The details of all staple strand DNA sequences are listed in Table S2.

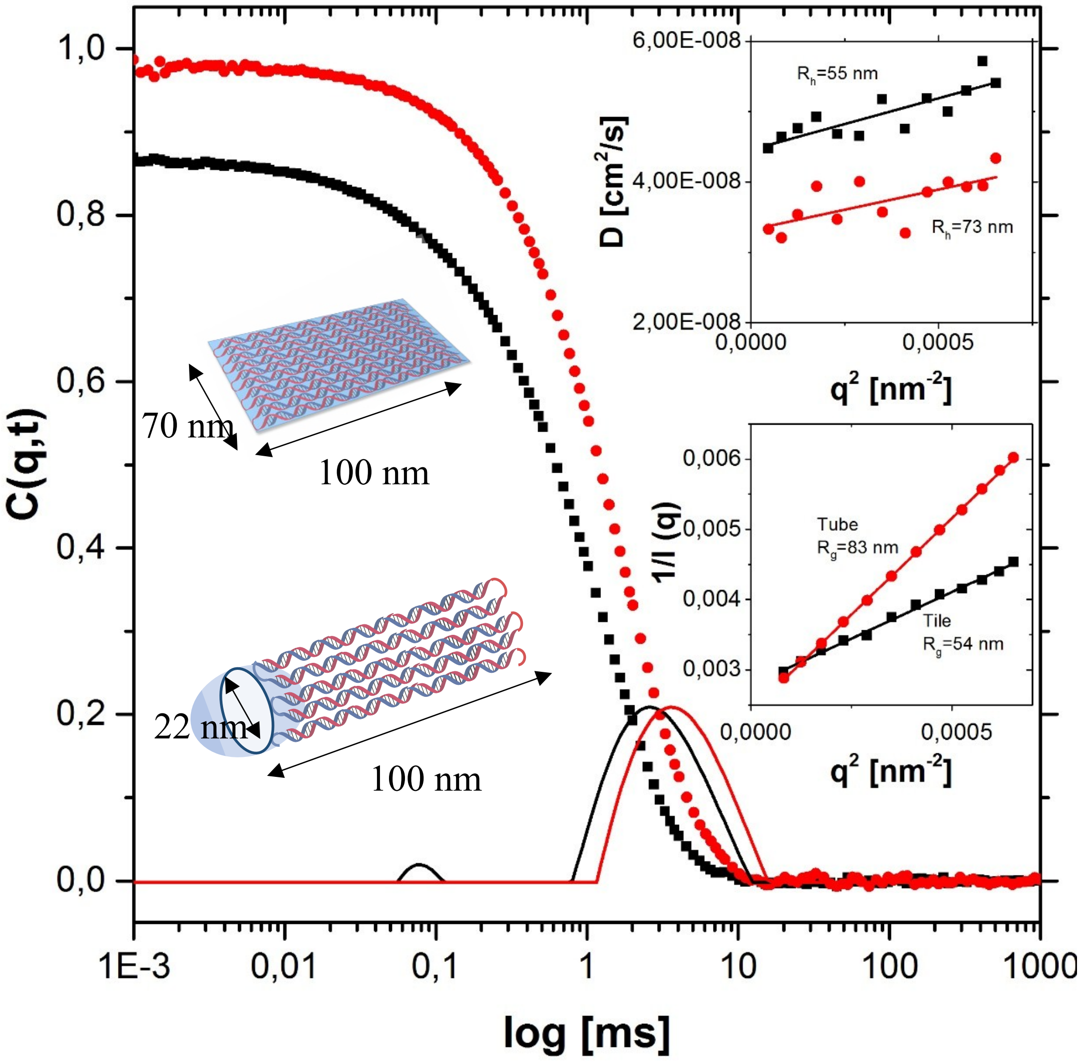

Figure S2 Relaxation functions $C_{vv}(q,t)$ for the translational diffusion dynamics in aqueous solution of the DNA tile (black filled squares) and DNA tube (red filled circles) at 20°C at a scattering wave vector (q=0.009 nm-1). Inverse Laplace transformation of experimental data yielded the distribution of one population for both the DNA tile and the DNA tube. Upper inset: The diffusion coefficient D vs q^2, R_h (tile) = 55 nm and R_h(tube) = 73 nm. Lower inset: Light scattering intensity 1/I(q) as a function of q^2 for the DNA tile (black squares) and the DNA tube (red circles). R_g(tile)=54 nm R_g(tube) = 83 nm.

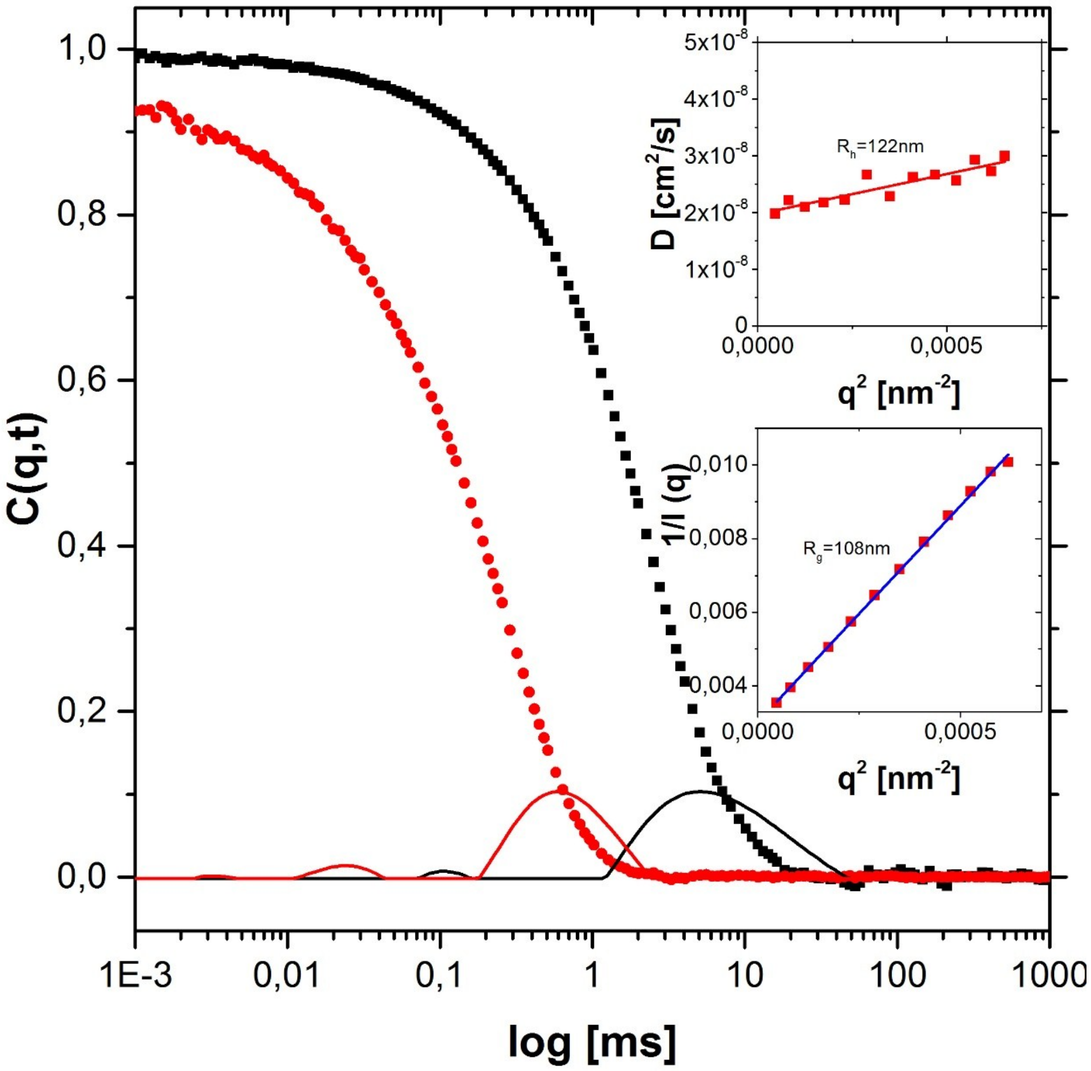

Figure S3 Normalized field correlation functions $C_{vv}(q,t)$ at a scattering wave vector q=0.009 nm^{-1} (black filled squares) and q=0.024 nm^{-1} (red filled circles) for the translational diffusion dynamics in aqueous solution of polymer coated DNA tube at 20°C. Inverse Laplace transformation of experimental data yielded to distribution of two populations for both wave vectors. Upper right inset: Double logarithmic plot of the diffusion coefficient D, R_h=122 nm. Lower right inset: 1/I(q) versus q^2 for the polymer coated DNA tube (black squares). From equation 1, R_g was calculated. R_g=108 nm.

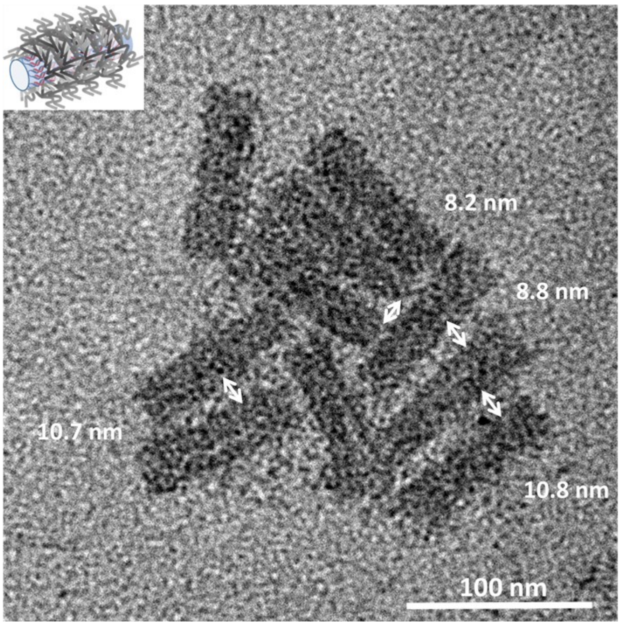

Figure S4 TEM image of stacking polymer tube.

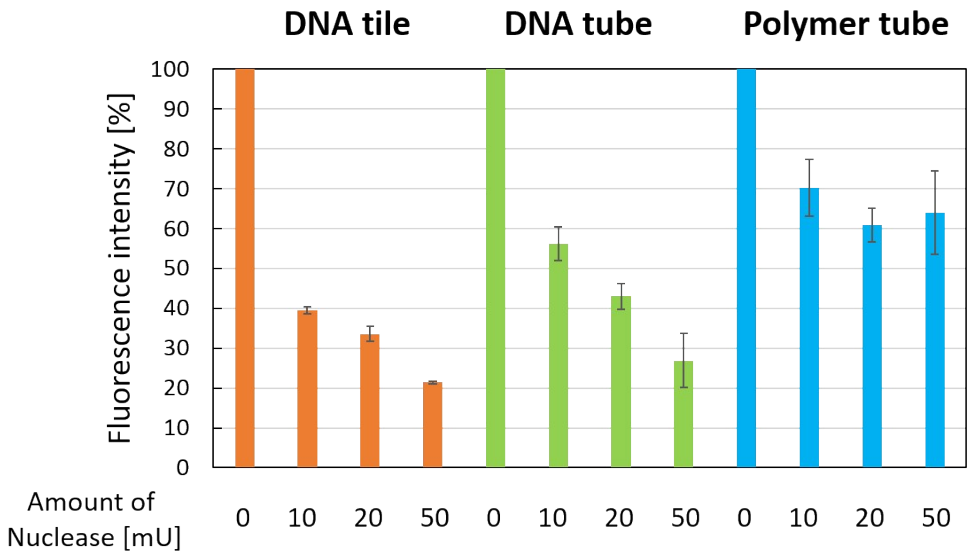

Figure S5 Stability of different DNA origami structures against nuclease digestion. DNA tile, tube, and polymer tube were labeled with SYBR-safe for 30 min. SYBR safe is a cyanine-based organic dye, which shows high fluorescence signal when it is intercalated into dsDNA. Thus, the degradation of DNA origami causes SYBR safe release from DNA origami resulting in decrease of the florescence intensity. Different amounts of nuclease (0-50 mU) were added to the labeled DNA origami structures and incubated at 37 degrees for 30 min. The fluorescence intensity of SYBR-safe was recorded and plotted as fluorescence intensity compared to the non-nuclease treated sample (the columns with amount of Dnase "0"). Since both ends of the DNA tubes are open, nucleases could in principle access the tube from both ends, which might explain the 30 % decrease of fluorescence intensity. However, after polymer coating, about 60 % to 70 % emission was observed for the polymer tube, compared to the DNA tube, for which only 20 % to 30 % emission intensity was recorded.

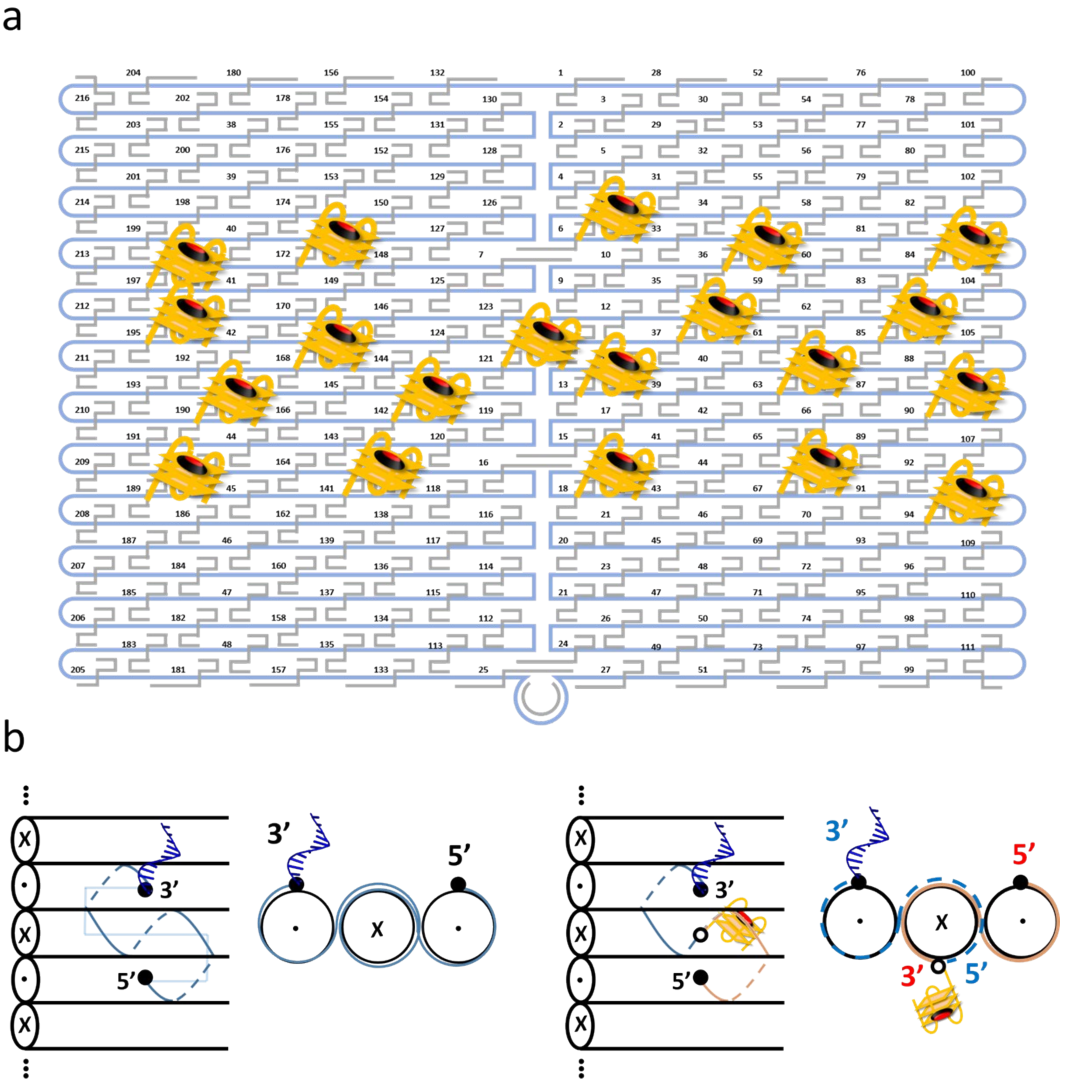

Figure S6 DNAzyme-incorporated DNA tile. (a) 20 DNAzyme moieties are positioned onto the surface opposite to DNA handle-introduced surface (Figure S1). (b) DNA handles are introduced to staple DNA sequence by extending its 3' that are exposed on to the surface (left). To introduce DNAzyme to the opposite side to DNA handle, DNA handle extended sequences (blue, left) are divided into two sequences; DNA handle-extended part (dashed blue, right) and DNAzyme incorporated part (orange, right).

Table S1 Summary of dimensions of the DNA tile, DNA tube, polymer tube, G4-incorporated DNA tube before / after ATRP (G4-tube / G4-polymer tube) from theoretical, AFM, and DLS.

Construct		Theoretical (nm)	AFM (nm)	DLS, R_h (nm)
DNA Tile	L	100	99.0 ± 2.2	55±3
	W	70	78.0 ± 4.0	
	H	2	3.1 ± 0.1	
DNA Tube	L	100	97.0 ± 4.9	83 ±2
	W	22	36.0 ± 6.0	
	H	22	5.0 ± 0.7	
Polymer Tube		-	91.0 ± 6.4	122±13
			44.0 ± 6.0	
			7.0 ± 0.5	
G4-Tube	L	100	93.3 ± 3.9	
	W	22	37.0 ± 4.0	
	H	22	7.2 ± 1.0	
G4-Polymer Tube		-	95.7 ± 5.7	
			55.0 ± 10	
			11.1 ± 1.9	

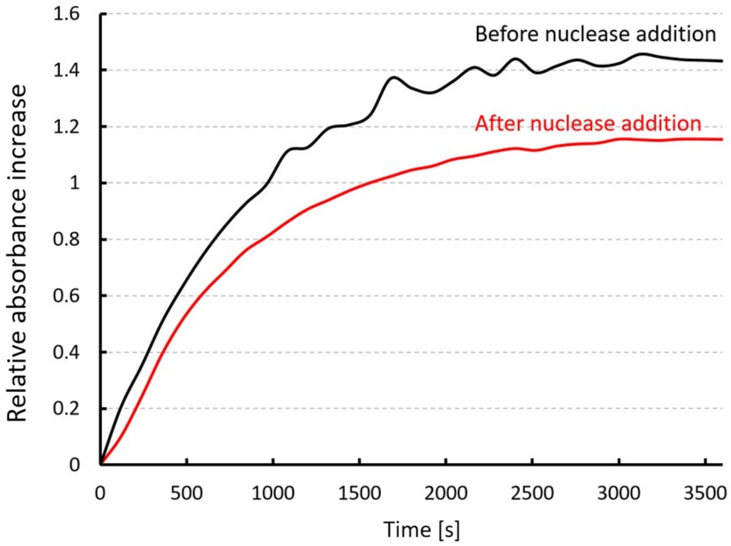

Figure S7 ABTS activity of DNAzymes in polymer tubes before / after nuclease addition (50 mU). 80 % of the DNAzyme activity was maintained even in nuclease presence.

Table S2 Detail of staple DNA sequences. Each number corresponds to the position shown in Figure S1. The Sequences extended with sticky DNA handle sequence at 3' are named as "3stX (X = position number)". Folding DNA sequences to transform DN tile to DNA tube are named as "FX". For preparing DNAzyme-introduce DNA tile, the sequences are separated into DNA handle-extended part (3stX half) and DNAzyme-extended part (g4-X half)

No.	Sequence
1	CAAGCCCAATAGGAACCCATGTACAAACAGTT
3st2	AATGCCCCGTAACAGTGCCCGTATCTCCCTCATTTTTTTAGTAGGTGGTAGAG
3st3	TGCCTTGACTGCCTATTTCGGAACAGGGATAGTTTTTTTAGTAGGTGGTAGAG
3st4	GAGCCGCCCCACCACCGGAACCGCGACGGAAATTTTTTTAGTAGGTGGTAGAG
3st5	AACCAGAGACCCTCAGAACCGCCAGGGGTCAGTTTTTTTAGTAGGTGGTAGAG
3st6	TTATTCATAGGGAAGGTAAATATTCATTCAGTTTTTTTTAGTAGGTGGTAGAG
3st7	CATAACCCGAGGCATAGTAAGAGCTTTTTAAGTTTTTTTAGTAGGTGGTAGAG
3st8	ATTGAGGGTAAAGGTGAATTATCAATCACCGGTTTTTTTAGTAGGTGGTAGAG
3st9	AAAAGTAATATCTTACCGAAGCCCTTCCAGAGTTTTTTTAGTAGGTGGTAGAG
3st10	GCAATAGCGCAGATAGCCGAACAATTCAACCGTTTTTTTAGTAGGTGGTAGAG
3st11	CCTAATTTACGCTAACGAGCGTCTAATCAATATTTTTTTAGTAGGTGGTAGAG
3st12	TCTTACCAGCCAGTTACAAAATAAATGAAATATTTTTTTAGTAGGTGGTAGAG
3st13	ATCGGCTGCGAGCATGTAGAAACCTATCATATTTTTTTTAGTAGGTGGTAGAG
3st14	CTAATTTATCTTTCCTTATCATTCATCCTGAATTTTTTTAGTAGGTGGTAGAG
3st15	GCGTTATAGAAAAAGCCTGTTTAGAAGGCCGGTTTTTTTAGTAGGTGGTAGAG
3st16	GCTCATTTTCGCATTAAATTTTTGAGCTTAGATTTTTTTAGTAGGTGGTAGAG
3st17	AATTACTACAAATTCTTACCAGTAATCCCATCTTTTTTTAGTAGGTGGTAGAG
3st18	TTAAGACGTTGAAAACATAGCGATAACAGTACTTTTTTTAGTAGGTGGTAGAG
3st19	TAGAATCCCTGAGAAGAGTCAATAGGAATCATTTTTTTTAGTAGGTGGTAGAG
3st20	CTTTTACACAGATGAATATACAGTAAACAATTTTTTTTTAGTAGGTGGTAGAG
3st21	TTTAACGTTCGGGAGAAACAATAATTTTCCCTTTTTTTTAGTAGGTGGTAGAG
3st22	CGACAACTAAGTATTAGACTTTACAATACCGATTTTTTTAGTAGGTGGTAGAG
3st23	GGATTTAGCGTATTAAATCCTTTGTTTTCAGGTTTTTTTAGTAGGTGGTAGAG
3st24	ACGAACCAAAACATCGCCATTAAATGGTGGTTTTTTTTTAGTAGGTGGTAGAG
25	GAACGTGGCGAGAAAGGAAGGGAACAAACTAT
3st26	TAGCCCTACCAGCAGAAGATAAAAACATTTGATTTTTTTAGTAGGTGGTAGAG

27	CGGCCTTGCTGGTAATATCCAGAACGAACTGA
28	CTCAGAGCCACCACCCTCATTTTCCTATTATT
3st29	CTGAAACAGGTAATAAGTTTTAACCCCTCAGATTTTTTTAGTAGGTGGTAGAG
3st30	AGTGTACTTGAAAGTATTAAGAGGCCGCCACCTTTTTTTAGTAGGTGGTAGAG
3st31	GCCACCACTCTTTTCATAATCAAACCGTCACCTTTTTTTAGTAGGTGGTAGAG
3st32	GTTTGCCACCTCAGAGCCGCCACCGATACAGGTTTTTTTAGTAGGTGGTAGAG
3st33	GACTTGAGAGACAAAAGGGCGACAAGTTACCATTTTTTTAGTAGGTGGTAGAG
3st34	AGCGCCAACCATTTGGGAATTAGATTATTAGCTTTTTTTAGTAGGTGGTAGAG
3st35	GAAGGAAAATAAGAGCAAGAAACAACAGCCATTTTTTTTAGTAGGTGGTAGAG
3st36	GCCCAATACCGAGGAAACGCAATAGGTTTACCTTTTTTTAGTAGGTGGTAGAG
3st37	ATTATTTAACCCAGCTACAATTTTCAAGAACGTTTTTTTAGTAGGTGGTAGAG
3st38	TATTTTGCTCCCAATCCAAATAAGTGAGTTAATTTTTTTAGTAGGTGGTAGAG
3st39	GGTATTAAGAACAAGAAAAATAATTAAAGCCATTTTTTTAGTAGGTGGTAGAG
3st40	TAAGTCCTACCAAGTACCGCACTCTTAGTTGCTTTTTTTAGTAGGTGGTAGAG
3st41	ACGCTCAAAATAAGAATAAACACCGTGAATTTTTTTTTTAGTAGGTGGTAGAG
3st42	AGGCGTTACAGTAGGGCTTAATTGACAATAGATTTTTTTAGTAGGTGGTAGAG
3st43	ATCAAAATCGTCGCTATTAATTAACGGATTCGTTTTTTTAGTAGGTGGTAGAG
3st44	CTGTAAATCATAGGTCTGAGAGACGATAAATATTTTTTTAGTAGGTGGTAGAG
3st45	CCTGATTGAAAGAAATTGCGTAGACCCGAACGTTTTTTTAGTAGGTGGTAGAG
3st46	ACAGAAATCTTTGAATACCAAGTTCCTTGCTTTTTTTTTAGTAGGTGGTAGAG
3st47	TTATTAATGCCGTCAATAGATAATCAGAGGTGTTTTTTTAGTAGGTGGTAGAG
3st48	AGATTAGATTTAAAAGTTTGAGTACACGTAAATTTTTTTAGTAGGTGGTAGAG
3st49	AGGCGGTCATTAGTCTTTAATGCGCAATATTATTTTTTTAGTAGGTGGTAGAG
3st50	GAATGGCTAGTATTAACACCGCCTCAACTAATTTTTTTTAGTAGGTGGTAGAG
51	CCGCCAGCCATTGCAACAGGAAAAATATTTTT
52	CCCTCAGAACCGCCACCCTCAGAACTGAGACT
3st53	CCTCAAGAATACATGGCTTTTGATAGAACCACTTTTTTTAGTAGGTGGTAGAG
3st54	TAAGCGTCGAAGGATTAGGATTAGTACCGCCATTTTTTTAGTAGGTGGTAGAG
3st 55	CACCAGAGTTCGGTCATAGCCCCCGCCAGCAATTTTTTTAGTAGGTGGTAGAG
3st 56	TCGGCATTCCGCCGCCAGCATTGACGTTCCAGTTTTTTTAGTAGGTGGTAGAG
3st 57	AATCACCAAATAGAAAATTCATATATAACGGATTTTTTTAGTAGGTGGTAGAG
3st 58	TCACAATCGTAGCACCATTACCATCGTTTTCATTTTTTTAGTAGGTGGTAGAG
3st 59	ATACCCAAGATAACCCACAAGAATAAACGATTTTTTTTTAGTAGGTGGTAGAG
3st60	ATCAGAGAAAGAACTGGCATGATTTTATTTTGTTTTTTTAGTAGGTGGTAGAG

3st61	TTTTGTTTAAGCCTTAAATCAAGAATCGAGAATTTTTTTAGTAGGTGGTAGAG
3st62	AGGTTTTGAACGTCAAAAATGAAAGCGCTAATTTTTTTTAGTAGGTGGTAGAG
3st63	CAAGCAAGACGCGCCTGTTTATCAAGAATCGCTTTTTTTAGTAGGTGGTAGAG
3st64	AATGCAGACCGTTTTTATTTTCATCTTGCGGGTTTTTTTAGTAGGTGGTAGAG
3st65	CATATTTAGAAATACCGACCGTGTTACCTTTTTTTTTTTAGTAGGTGGTAGAG
3st66	AATGGTTTACAACGCCAACATGTAGTTCAGCTTTTTTTTAGTAGGTGGTAGAG
3st67	TAACCTCCATATGTGAGTGAATAAACAAAATCTTTTTTTAGTAGGTGGTAGAG
3st68	AAATCAATGGCTTAGGTTGGGTTACTAAATTTTTTTTTTAGTAGGTGGTAGAG
3st 69	GCGCAGAGATATCAAAATTATTTGACATTATCTTTTTTTAGTAGGTGGTAGAG
3st 70	AACCTACCGCGAATTATTCATTTCCAGTACATTTTTTTTAGTAGGTGGTAGAG
3st 71	ATTTTGCGTCTTTAGGAGCACTAAGCAACAGTTTTTTTTAGTAGGTGGTAGAG
3st 72	CTAAAATAGAACAAAGAAACCACCAGGGTTAGTTTTTTTAGTAGGTGGTAGAG
3st73	GCCACGCTATACGTGGCACAGACAACGCTCATTTTTTTTAGTAGGTGGTAGAG
3st74	GCGTAAGAGAGAGCCAGCAGCAAAAAGGTTATTTTTTTTAGTAGGTGGTAGAG
75	GGAAATACCTACATTTTGACGCTCACCTGAAA
76	TATCACCGTACTCAGGAGGTTTAGCGGGGTTT
3st77	TGCTCAGTCAGTCTCTGAATTTACCAGGAGGTTTTTTTAGTAGGTGGTAGAG
3st78	GGAAAGCGACCAGGCGGATAAGTGAATAGGTGTTTTTTTAGTAGGTGGTAGAG
3st79	TGAGGCAGGCGTCAGACTGTAGCGTAGCAAGGTTTTTTTAGTAGGTGGTAGAG
3st80	TGCCTTTAGTCAGACGATTGGCCTGCCAGAATTTTTTTTAGTAGGTGGTAGAG
3st81	CCGGAAACACACCACGGAATAAGTAAGACTCCTTTTTTTAGTAGGTGGTAGAG
3st82	ACGCAAAGGTCACCAATGAAACCAATCAAGTTTTTTTTTAGTAGGTGGTAGAG
3st83	TTATTACGGTCAGAGGGTAATTGAATAGCAGCTTTTTTTAGTAGGTGGTAGAG
3st84	TGAACAAACAGTATGTTAGCAAACTAAAAGAATTTTTTTAGTAGGTGGTAGAG
3st85	CTTTACAGTTAGCGAACCTCCCGACGTAGGAATTTTTTTAGTAGGTGGTAGAG
3st86	GAGGCGTTAGAGAATAACATAAAAGAACACCCTTTTTTTAGTAGGTGGTAGAG
3st87	TCATTACCCGACAATAAACAACATATTTAGGCTTTTTTTAGTAGGTGGTAGAG
3st88	CCAGACGAGCGCCCAATAGCAAGCAAGAACGCTTTTTTTAGTAGGTGGTAGAG
3st89	AGAGGCATAATTTCATCTTCTGACTATAACTATTTTTTTAGTAGGTGGTAGAG
3st90	TTTTAGTTTTTCGAGCCAGTAATAAATTCTGTTTTTTTTAGTAGGTGGTAGAG
3st91	TATGTAAACCTTTTTTAATGGAAAAATTACCTTTTTTTTAGTAGGTGGTAGAG
3st92	TTGAATTATGCTGATGCAAATCCACAAATATATTTTTTTAGTAGGTGGTAGAG
3st93	GAGCAAAAACTTCTGAATAATGGAAGAAGGAGTTTTTTTAGTAGGTGGTAGAG
3st94	TGGATTATGAAGATGATGAAACAAAATTTCATTTTTTTTAGTAGGTGGTAGAG

3st95	CGGAATTATTGAAAGGAATTGAGGTGAAAAATTTTTTTTAGTAGGTGGTAGAG
3st96	ATCAACAGTCATCATATTCCTGATTGATTGTTTTTTTTTAGTAGGTGGTAGAG
3st97	CTAAAGCAAGATAGAACCCTTCTGAATCGTCTTTTTTTTAGTAGGTGGTAGAG
3st98	GCCAACAGTCACCTTGCTGAACCTGTTGGCAATTTTTTTTAGTAGGTGGTAGAG
99	GAAATGGATTATTTACATTGGCAGACATTCTG
100	TTTTTATAAGTATAGCCCGGCCGTCGAG
101	AGGGTTGATTTTATAAATCCTCATTAAATGATATTC
102	ACAAACAATTTTAATCAGTAGCGACAGATCGATAGC
103	AGCACCGTTTTTTAAAGGTGGCAACATAGTAGAAAA
104	TACATACATTTTGACGGGAGAATTAACTACAGGGAA
105	GCGCATTATTTTGCTTATCCGGTATTCTAAATCAGA
106	TATAGAAGTTTTCGACAAAAGGTAAAGTAGAGAATA
107	TAAAGTACTTTTCGCGAGAAAACTTTTTATCGCAAG
108	ACAAAGAATTTTATTAATTACATTTAACACATCAAG
109	AAAACAAATTTTTTCATCAATATAATCCTATCAGAT
110	GATGGCAATTTTAATCAATATCTGGTCACAAATATC
111	AAACCCTCTTTTACCAGTAATAAAAGGGATTCACCAGTCACACGTTTT
3st112	CCGAAATCCGAAAATCCTGTTTGAAGCCGGAATTTTTTTTAGTAGGTGGTAGAG
3st113	CCAGCAGGGGCAAAATCCCTTATAAAGCCGGCTTTTTTTTAGTAGGTGGTAGAG
3st114	GCATAAAGTTCCACACAACATACGAAGCGCCATTTTTTTTAGTAGGTGGTAGAG
3st115	GCTCACAATGTAAAGCCTGGGGTGGGTTTGCCTTTTTTTTAGTAGGTGGTAGAG
3st116	TTCGCCATTGCCGGAAACCAGGCATTAAATCATTTTTTTTAGTAGGTGGTAGAG
3st117	GCTTCTGGTCAGGCTGCGCAACTGTGTTATCCTTTTTTTTAGTAGGTGGTAGAG
3st118	GTTAAAATTTTAACCAATAGGAACCCGGCACCTTTTTTTTAGTAGGTGGTAGAG
3st119	AGACAGTCATTCAAAAGGGTGAGAAGCTATATTTTTTTTTAGTAGGTGGTAGAG
3st120	AGGTAAAGAAATCACCATCAATATAATATTTTTTTTTTTAGTAGGTGGTAGAG
3st121	TTTCATTTGGTCAATAACCTGTTTATATCGCGTTTTTTTTAGTAGGTGGTAGAG
3st122	TCGCAAATGGGGCGCGAGCTGAAATAATGTGTTTTTTTTTAGTAGGTGGTAGAG
3st123	TTTTAATTGCCCGAAAGACTTCAAAACACTATTTTTTTTTAGTAGGTGGTAGAG
3st124	AAGAGGAACGAGCTTCAAAGCGAAGATACATTTTTTTTTTAGTAGGTGGTAGAG
3st125	GGAATTACTCGTTTACCAGACGACAAAAGATTTTTTTTTTAGTAGGTGGTAGAG
3st126	GAATAAGGACGTAACAAAGCTGCTCTAAAACATTTTTTTTAGTAGGTGGTAGAG
3st127	CCAAATCACTTGCCCTGACGAGAACGCCAAAATTTTTTTTAGTAGGTGGTAGAG
3st128	CTCATCTTGAGGCAAAAGAATACAGTGAATTTTTTTTTTTAGTAGGTGGTAGAG

3st129	AAACGAAATGACCCCCAGCGATTATTCATTACTTTTTTTAGTAGGTGGTAGAG
3st130	CTTAAACATCAGCTTGCTTTCGAGCGTAACACTTTTTTTAGTAGGTGGTAGAG
3st131	TCGGTTTAGCTTGATACCGATAGTCCAACCTATTTTTTTAGTAGGTGGTAGAG
132	TGAGTTTCGTCACCAGTACAAACTTAATTGTA
133	CCCCGATTTAGAGCTTGACGGGGAAATCAAAA
3st134	GAATAGCCGCAAGCGGTCCACGCTCCTAATGATTTTTTTAGTAGGTGGTAGAG
3st135	GAGTTGCACGAGATAGGGTTGAGTAAGGGAGCTTTTTTTAGTAGGTGGTAGAG
3st136	GTGAGCTAGTTTCCTGTGTGAAATTTGGGAAGTTTTTTTAGTAGGTGGTAGAG
3st137	TCATAGCTACTCACATTAATTGCGCCCTGAGATTTTTTTAGTAGGTGGTAGAG
3st138	GGCGATCGCACTCCAGCCAGCTTTGCCATCAATTTTTTTAGTAGGTGGTAGAG
3st139	GAAGATCGGTGCGGGCCTCTTCGCAATCATGGTTTTTTTAGTAGGTGGTAGAG
3st140	AAATAATTTTAAATTGTAAACGTTGATATTCATTTTTTTAGTAGGTGGTAGAG
3st141	GCAAATATCGCGTCTGGCCTTCCTGGCCTCAGTTTTTTTAGTAGGTGGTAGAG
3st142	ACCGTTCTAAATGCAATGCCTGAGAGGTGGCATTTTTTTAGTAGGTGGTAGAG
3st143	TATATTTTAGCTGATAAATTAATGTTGTATAATTTTTTTAGTAGGTGGTAGAG
3st144	TCAATTCTTTTAGTTTGACCATTACCAGACCGTTTTTTTAGTAGGTGGTAGAG
3st145	CGAGTAGAACTAATAGTAGTAGCAAACCCTCATTTTTTTAGTAGGTGGTAGAG
3st146	GAAGCAAAAAAGCGGATTGCATCAGATAAAAATTTTTTTAGTAGGTGGTAGAG
3st147	TCAGAAGCCTCCAACAGGTCAGGATCTGCGAATTTTTTTAGTAGGTGGTAGAG
3st148	CCAAAATATAATGCAGATACATAAACACCAGATTTTTTTAGTAGGTGGTAGAG
3st149	CATTCAACGCGAGAGGCTTTTGCATATTATAGTTTTTTTAGTAGGTGGTAGAG
3st150	ACGAGTAGTGACAAGAACCGGATATACCAAGCTTTTTTTAGTAGGTGGTAGAG
3st151	AGTAATCTTAAATTGGGCTTGAGAGAATACCATTTTTTTAGTAGGTGGTAGAG
3st152	GCGAAACATGCCACTACGAAGGCATGCGCCGATTTTTTTAGTAGGTGGTAGAG
3st153	ATACGTAAAAGTACAACGGAGATTTCATCAAGTTTTTTTAGTAGGTGGTAGAG
3st154	CAATGACACTCCAAAAGGAGCCTTACAACGCCTTTTTTTAGTAGGTGGTAGAG
3st155	AAAAAAGGACAACCATCGCCCACGCGGGTAAATTTTTTTAGTAGGTGGTAGAG
156	TGTAGCATTCCACAGACAGCCCTCATCTCCAA
157	GTAAAGCACTAAATCGGAACCCTAGTTGTTCC
3st158	AGTTTGGAGCCCTTCACCGCCTGGTTGCGCTCTTTTTTTAGTAGGTGGTAGAG
3st159	AGCTGATTACAAGAGTCCACTATTGAGGTGCCTTTTTTTAGTAGGTGGTAGAG
3st160	ACTGCCCGCCGAGCTCGAATTCGTTATTACGCTTTTTTTAGTAGGTGGTAGAG
3st161	CCCGGGTACTTTCCAGTCGGGAAACGGGCAACTTTTTTTAGTAGGTGGTAGAG
3st162	CAGCTGGCGGACGACGACAGTATCGTAGCCAGTTTTTTTAGTAGGTGGTAGAG

3st163	GTTTGAGGGAAAGGGGGATGTGCTAGAGGATCTTTTTTTAGTAGGTGGTAGAG
3st164	CTTTCATCCCCAAAAACAGGAAGACCGGAGAGTTTTTTTAGTAGGTGGTAGAG
3st165	AGAAAAGCAACATTAAATGTGAGCATCTGCCATTTTTTTAGTAGGTGGTAGAG
3st166	GGTAGCTAGGATAAAAATTTTTAGTTAACATCTTTTTTTAGTAGGTGGTAGAG
3st167	CAACGCAATTTTTGAGAGATCTACTGATAATCTTTTTTTAGTAGGTGGTAGAG
3st168	CAATAAATACAGTTGATTCCCAATTTAGAGAGTTTTTTTAGTAGGTGGTAGAG
3st169	TCCATATACATACAGGCAAGGCAACTTTATTTTTTTTTTAGTAGGTGGTAGAG
3st170	TACCTTTAAGGTCTTTACCCTGACAAAGAAGTTTTTTTTAGTAGGTGGTAGAG
3st171	CAAAAATCATTGCTCCTTTTGATAAGTTTCATTTTTTTTAGTAGGTGGTAGAG
3st172	TTTGCCAGATCAGTTGAGATTTAGTGGTTTAATTTTTTTAGTAGGTGGTAGAG
3st173	AAAGATTCAGGGGGTAATAGTAAACCATAAATTTTTTTTAGTAGGTGGTAGAG
3st174	TTTCAACTATAGGCTGGCTGACCTTGTATCATTTTTTTTAGTAGGTGGTAGAG
3st175	CCAGGCGCTTAATCATTGTGAATTACAGGTAGTTTTTTTAGTAGGTGGTAGAG
3st176	CGCCTGATGGAAGTTTCCATTAAACATAACCGTTTTTTTAGTAGGTGGTAGAG
3st177	TTTCATGAAAATTGTGTCGAAATCTGTACAGATTTTTTTAGTAGGTGGTAGAG
3st178	ATATATTCTTTTTTCACGTTGAAAATAGTTAGTTTTTTTAGTAGGTGGTAGAG
3st179	AATAATAAGGTCGCTGAGGCTTGCAAAGACTTTTTTTTTAGTAGGTGGTAGAG
180	CGTAACGATCTAAAGTTTTGTCGTGAATTGCG
181	ACCCAAATCAAGTTTTTTGGGGTCAAAGAACG
3st182	TGGACTCCCTTTTCACCAGTGAGACCTGTCGTTTTTTTTAGTAGGTGGTAGAG
3st183	TGGTTTTTAACGTCAAAGGGCGAAGAACCATCTTTTTTTAGTAGGTGGTAGAG
3st184	GCCAGCTGCCTGCAGGTCGACTCTGCAAGGCGTTTTTTTAGTAGGTGGTAGAG
3st185	CTTGCATGCATTAATGAATCGGCCCGCCAGGGTTTTTTTAGTAGGTGGTAGAG
3st186	ATTAAGTTCGCATCGTAACCGTGCGAGTAACATTTTTTTAGTAGGTGGTAGAG
3st187	TAGATGGGGGGTAACGCCAGGGTTGTGCCAAGTTTTTTTAGTAGGTGGTAGAG
3st188	ACCCGTCGTCATATGTACCCCGGTAAAGGCTATTTTTTTAGTAGGTGGTAGAG
3st189	CATGTCAAGATTCTCCGTGGGAACCGTTGGTGTTTTTTTAGTAGGTGGTAGAG
3st190	TCAGGTCACTTTTGCGGGAGAAGCAGAATTAGTTTTTTTAGTAGGTGGTAGAG
3st191	CTGTAATATTGCCTGAGAGTCTGGAAAACTAGTTTTTTTAGTAGGTGGTAGAG
3st192	CAAAATTAAAGTACGGTGTCTGGAAGAGGTCATTTTTTTAGTAGGTGGTAGAG
3st193	TGCAACTAAGCAATAAAGCCTCAGTTATGACCTTTTTTTAGTAGGTGGTAGAG
3st194	TTTTTGCGCAGAAAACGAGAATGAATGTTTAGTTTTTTTAGTAGGTGGTAGAG
3st195	AAACAGTTGATGGCTTAGAGCTTATTTAAATATTTTTTTAGTAGGTGGTAGAG
3st196	ACTGGATAACGGAACAACATTATTACCTTATGTTTTTTTAGTAGGTGGTAGAG

3st197	ACGAACTAGCGTCCAATACTGCGGAATGCTTTTTTTTTTAGTAGGTGGTAGAG
3st198	CGATTTTAGAGGACAGATGAACGGCGCGACCTTTTTTTTAGTAGGTGGTAGAG
3st199	CTTTGAAAAGAACTGGCTCATTATTTAATAAATTTTTTTAGTAGGTGGTAGAG
3st200	GCTCCATGAGAGGCTTTGAGGACTAGGGAGTTTTTTTTAGTAGGTGGTAGAG
3st201	ACGGCTACTTACTTAGCCGGAACGCTGACCAATTTTTTTAGTAGGTGGTAGAG
3st202	AAAGGCCGAAAGGAACAACTAAAGCTTTCCAGTTTTTTTAGTAGGTGGTAGAG
3st203	GAGAATAGCTTTTGCGGGATCGTCGGGTAGCATTTTTTTAGTAGGTGGTAGAG
204	ACGTTAGTAAATGAATTTTCTGTAAGCGGAGT
205	TTTTCGATGGCCCACTACGTAAACCGTC
206	TATCAGGGTTTTCGGTTTGCGTATTGGGAACGCGCG
207	GGGAGAGGTTTTTGTAAAACGACGGCCATTCCCAGT
208	CACGACGTTTTTGTAATGGGATAGGTCAAAACGGCG
209	GATTGACCTTTTGATGAACGGTAATCGTAGCAAACA
210	AGAGAATCTTTTGGTTGTACCAAAAACAAGCATAAA
211	GCTAAATCTTTTCTGTAGCTCAACATGTATTGCTGA
212	ATATAATGTTTTCATTGAATCCCCCTCAAATCGTCA
213	TAAATATTTTTTGGAAGAAAAATCTACGACCAGTCA
214	GGACGTTGTTTTTCATAAGGGAACCGAAAGGCGCAG
215	ACGGTCAATTTTGACAGCATCGGAACGAACCCTCAG
216	CAGCGAAAATTTTACTTTCAACAGTTTCTGGGATTTTGCTAAACTTTT
217	AACATCACTTGCCTGAGTAGAAGAACT
218	TGTAGCAATACTTCTTTGATTAGTAAT
219	AGTCTGTCCATCACGCAAATTAACCGT
220	ATAATCAGTGAGGCCACCGAGTAAAAG
221	ACGCCAGAATCCTGAGAAGTGTTTTT
222	TTAAAGGGATTTTAGACAGGAACGGT
223	AGAGCGGGAGCTAAACAGGAGGCCGA
224	TATAACGTGCTTTCCTCGTTAGAATC
225	GTACTATGGTTGCTTTGACGAGCACG
226	GCGCTTAATGCGCCGCTACAGGGCGC
F1	AATAATAATAATAATCAAGCCCAATAGGAACCCATGTACAAACAGTT
F25	AATAATAATAATAATGAACGTGGCGAGAAAGGAAGGGAACAAACTAT
F27	CAAGCCCACTGGTAATATCCAGAACGAACTGA
F28	CCGCCAGCCACCACCCTCATTTTCCTATTATT

F51	CTCAGAGCCATTGCAACAGGAAAAATATTTTT
F52	GGAAATACACCGCCACCCTCAGAACTGAGACT
F75	CCCTCAGACTACATTTTGACGCTCACCTGAAA
F76	GAAATGGATACTCAGGAGGTTTAGCGGGGTTT
F99	TATCACCGTTATTTACATTGGCAGACATTCTG
F132	GAACGTGGGTCACCAGTACAAACTTAATTGTA
F133	TGTAGCATTAGAGCTTGACGGGGAAATCAAAA
F156	CCCCGATTTCCACAGACAGCCCTCATCTCCAA
F157	CGTAACGACTAAATCGGAACCCTAGTTGTTCC
F180	GTAAAGCATCTAAAGTTTTGTCGTGAATTGCG
F181	ACGTTAGTCAAGTTTTTTGGGGTCAAAGAACG
F204	ACCCAAATAAATGAATTTTCTGTAAGCGGAGT
g4-6half	TTATTCATAGGGAAGG TTTTGGGTAGGGCGGGTTGGG
3st6half	TAAATATT CATTCAGT TTTTTTTAGTAGGTGGTAGAG
g4-13half	ATCGGCTGCGAGCATG TTTTGGGTAGGGCGGGTTGGG
3st13half	TAGAAACCTATCATAT TTTTTTTAGTAGGTGGTAGAG
g4-18half	TTAAGACGTTGAAAAC TTTTGGGTAGGGCGGGTTGGG
3st18half	ATAGCGATAACAGTAC TTTTTTTAGTAGGTGGTAGAG
g4-36half	GCCCAATACCGAGGAA TTTTGGGTAGGGCGGGTTGGG
3st36half	ACGCAATAGGTTTACCTTTTTTTAGTAGGTGGTAGAG
g4-37half	ATTATTTAACCCAGCT TTTTGGGTAGGGCGGGTTGGG
3st37half	ACAATTTTCAAGAACG TTTTTTTAGTAGGTGGTAGAG
g4-63half	CAAGCAAGACGCGCCT TTTTGGGTAGGGCGGGTTGGG
3st63half	GTTTATCAAGAATCGC TTTTTTTAGTAGGTGGTAGAG
g4-67half	TAACCTCCATATGTGA TTTTGGGTAGGGCGGGTTGGG
3st67half	GTGAATAAACAAAATCTTTTTTTTAGTAGGTGGTAGAG
g4-84half	TGAACAAACAGTATGT TTTTGGGTAGGGCGGGTTGGG
3st84half	TAGCAAACTAAAAGAA TTTTTTTAGTAGGTGGTAGAG
g4-85half	CTTTACAGTTAGCGAA TTTTGGGTAGGGCGGGTTGGG
3st85half	CCTCCCGACGTAGGAA TTTTTTTAGTAGGTGGTAGAG
g4-90half	TTTTAGTTTTTCGAGC TTTTGGGTAGGGCGGGTTGGG
3st90half	CAGTAATAAATTCTGT TTTTTTTAGTAGGTGGTAGAG
g4-94half	TGGATTATGAAGATGA TTTTGGGTAGGGCGGGTTGGG
3st94half	TGAAACAAAATTTCAT TTTTTTTAGTAGGTGGTAGAG
g4-121half	TTTCATTTGGTCAATA TTTTGGGTAGGGCGGGTTGGG

3st121half	ACCTGTTTATATCGCGTTTTTTTAGTAGGTGGTAGAG
g4-141half	GCAAATATCGCGTCTG TTTTGGGTAGGGCGGGTTGGG
3st141half	GCCTTCCTGGCCTCAGTTTTTTTTAGTAGGTGGTAGAG
g4-142half	ACCGTTCTAAATGCAA TTTTGGGTAGGGCGGGTTGGG
3st142half	TGCCTGAGAGGTGGCA TTTTTTTAGTAGGTGGTAGAG
g4-168half	CAATAAATACAGTTGA TTTTGGGTAGGGCGGGTTGGG
3st168half	TTCCCAATTTAGAGAG TTTTTTTAGTAGGTGGTAGAG
g4-172half	TTTGCCAGATCAGTTG TTTTGGGTAGGGCGGGTTGGG
3st172half	AGATTTAGTGGTTTAA TTTTTTTAGTAGGTGGTAGAG
g4-189half	CATGTCAAGATTCTCC TTTTGGGTAGGGCGGGTTGGG
3st189half	GTGGGAACCGTTGGTG TTTTTTTAGTAGGTGGTAGAG
g4-190half	TCAGGTCACTTTTGCG TTTTGGGTAGGGCGGGTTGGG
3st190half	GGAGAAGCAGAATTAG TTTTTTTAGTAGGTGGTAGAG
g4-195half	AAACAGTTGATGGCTT TTTTGGGTAGGGCGGGTTGGG
3st195half	AGAGCTTATTTAAATA TTTTTTTAGTAGGTGGTAGAG
g4-199half	CTTTGAAAAGAACTGG TTTTGGGTAGGGCGGGTTGGG
3st199half	CTCATTATTTAATAAA TTTTTTTAGTAGGTGGTAGAG

References

[1] E. Stahl, T. G. Martin, F. Praetorius, H. Dietz, *Angew. Chem.Int. Ed.* 2014, 53, 12735-12740.

[2] S. E. Averick, S. K. Dey, D. Grahacharya, K. Matyjaszewski, S. R. Das, *Angew. Chem. Int. Ed.* 2014, 53, 2739-2744.

[3] P. W. Rothemund, *Nature* 2006, 440, 297-302

[4] S. M. Douglas, A. H. Marblestone, S. Teerapittayanon, A. Vazquez, G. M. Church, W. M. Shih, *Nucleic Acids Res.* 2009, 37, 5001–5006
